# Source Apportionment and Health Risk Assessment of Heavy Metals in Soils of Old Industrial Areas—A Case Study of Shanghai, China

**DOI:** 10.3390/ijerph20032395

**Published:** 2023-01-29

**Authors:** Chuan-Zheng Yuan, Xiang-Rong Wang

**Affiliations:** Department of Environmental Science and Engineering, Fudan University, Shanghai 200438, China

**Keywords:** GIS, health risk, heavy metal, PCA, spatial distribution

## Abstract

Heavy metals in the soil of industrial areas pose severe health risks to humans after land-use properties are transformed into residential land. The public exposure time and frequency will soar significantly under residential land. However, much uncertainty still exists about the relationship between soil heavy metal pollution and—human health risks in an old industrial zone in Shanghai, China. Principal component analysis—(PCA) was used to explore the main sources of these heavy metals. Kriging interpolation was u-sed to identify their spatial distribution and high-risk areas, and the Human Health risk model was used to measure health risk. The results illustrate that the pollution levels of Cd, Hg, and Pb in industrial land are more serious than those in irrigation cropland. Meanwhile, the results of PCA showed that there were two main pollution sources under irrigated cropland, a natural source and a traffic source, accounting for 44.1% and 31.0%, respectively, and there were three main pollution sources under industrial land, with natural sources accounting for 28.5%, traffic sources accounting for 25.7%, and industrial sources accounting for 13.1%. In addition, the health risk assessment results indicated that the priority control pollutants of non-carcinogenic risk and carcinogenic risk were Zn and Cr, respectively. The high-risk area was mainly located in the middle of the study area. These results indicate that eliminating heavy metal pollution in the soil of the industrial area is so important to decrease health risks. The results of this study provide theoretical contributions to early warning of health risks related to heavy metal pollution in industrial area soil and serve as a practical reference for speeding up the formulation of industrial land pollution management policies.

## 1. Introduction

Heavy metal (HM) pollution in the environment mainly comes from anthropogenic activities, such as mining, the discharge of industrial waste gas and wastewater, automobile exhaust, and the utilization of pesticides and fertilizers [1]. Heavy metals are highly toxic, long lasting, and difficult to biodegrade [2,3,4]. Once these substances accumulate in the environment, they will be a severe pollutant source that threatens ecology and human health safety [5]. For example, excessive As can cause cardiovascular diseases [6], and excessive Cd can lead to liver disease [7]. Pb poisoning causes 0.9 million deaths globally every year [8]. The issue of heavy metal pollution in soil has attracted attention from the world [9], especially in industrial areas [10].

Heavy metal pollution under different land uses has different effects. Gupta et al. [11] studied heavy metal pollution in agricultural land and found that the concentrations of Cd and Zn were high due to the use of pesticides and fertilizers. Peng et al. [12] studied the health risks of heavy metals in industrial land, and found that Pb, Cd, Zn, Ni, and As had a higher risk. Yuan and Wang [13] studied the risk of heavy metals under two land types and found that the risk of industrial land was higher. The reason why the risk of heavy metals varies under different land-use types is that the sources of pollution are different. Previous studies have focused on the pollution risk of heavy metals under specific land-use types without considering the impact of land-use change. For example, when industrial land is converted into residential land, pollution is caused by industrial emissions, but the time and frequency of exposure to residential land will increase, which will significantly amplify the health risks caused by heavy metals.

As the largest city in southern China, Shanghai has become one of the most economically developed and densely populated regions in China. Over the past few decades, many lands in Putuo have been utilized for industrial activities to promote industrial development [14]. HMs in the soils of the Putuo industrial area have become a major pollution problem, resulting in serious land degradation, and threatening sustainable land management [15]. To our knowledge, many previous studies in Shanghai on soil HMs have focused only on pollution evaluation, while few studies have focused on the health risk and source identification of HMs. In these directions, bi [16] studied the impact of Pb pollution on agriculture in Shanghai’s industrial zone but did not consider the influence of land-use covers on the results of health risk assessment in soil. As, Cr, Cd, Ni, Hg, Pb, Cu, and Zn are the basic pollutants in the soil environmental standards of China, excessive amounts will cause carcinogenic and non-carcinogenic risks to human health [17].

Previous studies on soil environmental risks in industrial areas mainly focused on the health impact on workers [18,19], but the exposure time and frequency of this impact cannot be compared with the impact on residential land. As an industrial output value of more than 1 trillion ¥ in 2021 [20], Shanghai ranks first in China and is the most important industrial city. With the change in urban planning, a large number of industrial land will be converted into residential land. However, at present, few studies have considered the impact of industrial pollution on residents under this change. Therefore, this study aims at more advanced ways to determine pollution sources and human health risks of eight heavy metals in the soil of the Putuo Industrial area in Shanghai. The specific objectives of this study were as follows: (1) investigate the concentrations and spatial distribution characteristics of eight heavy metals (As, Cr, Cd, Ni, Hg, Pb, Cu, and Zn) in the soil of an industrial area, (2) analyze the differences of pollution sources under different land-use covers in soil, and (3) identify priority control pollutants and areas under different land-use covers based on health risk assessment. Understanding the health risk in industrial land offers additional information for programming and managing the limited land more effectively; it also gives a better suggestion for preventing and controlling heavy metal pollution from the sources at the same time.

## 2. Materials and Methods

### 2.1. Study Area

**Figure 1:**
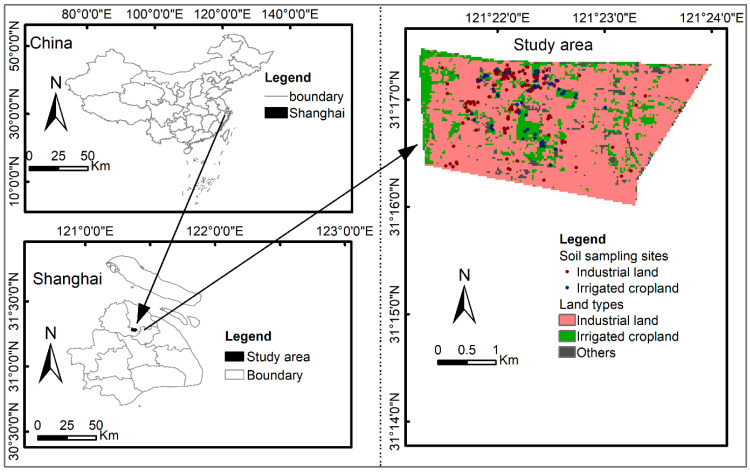
Location of the study area and distribution of soil sampling points.

The study area is located in northern Shanghai city, east China (Figure 1), with a total area of 4 km^2^ (121°21′1″–121°23′44″ E, 31°15′57″–31°17′27″ N). According to the 2010 China population survey, the total population of the study area is about 8.35 × 10^4^, with children accounting for 7.7% and adults accounting for 92.3%. The study area is influenced by a subtropical monsoon climate. The temperature of this region is 5.5–34.3 °C, and the average annual precipitation is 1200 mm. The main soil type in the study area is clay. At the end of the 20th century, the research area was still undeveloped and belonged to the suburbs, dominated by wasteland and farmland. At present, Shanghai, where the study area is located, is an important heavy industry base in China. There are many industrial types in the region, including sludge landfills, thermal power plants, spice plants, chemical plants, pharmaceutical plants, spice plants, ink plants, and wastewater treatment plants. Heavy metals are widespread pollutants in the industrial area of Shanghai City; moreover, the overall pollution level is increasing gradually year by year. Since the government intends to convert the land in the study area into residential land and parks, detailed pollution investigations and risk assessments are necessary.

### 2.2. Sampling and Testing

A total of 199 soil samples were taken from the upper soil layer (0–50 cm) in 2016. Five random sub-samples from a nearby 5 m^2^ area were mingled, and 0.5 kg soil samples were collected in a polyethylene bag and then brought back to the laboratory from each sample site. For further analyses, the soil samples were air-dried naturally at room temperature, and after debris removal, they were ground in an agate mortar and passed through a 20-mesh (<0.84 mm) nylon sieve. Thereafter, the samples were further filtered using a 200-mesh (<0.074 mm) sieve. A typical concentrated acid mixture of HNO_3_-HF-HClO_4_ (with a volume ratio of 3:1:1, purchased from Sinopharm Chemical Reagent Inc., CO, Shanghai China) was used for the digestion of samples and stored in glass bottles. Then, we measured the heavy metal concentrations (As, Cd, Cr, Ni, Hg, Pb, Cu, and Zn) of soil samples by Inductively Coupled Plasma Mass Spectrometry (ICP-MS, Agilent 7900, Palo Alto, CA, USA). Analysis and Quality Assurance/Quality Control (QA/QC) met the requirements of the USEPA 6020B [21].

### 2.3. Pollution Evaluation

The index of geo-accumulation (Igeo), originally proposed by Müller [22], was first used to assess sediment pollution levels. It has since then been widely used to assess soil pollution status, compared with environmental background values.

$$I_{geo}=\log_{2}\left(\frac{C_{I}}{K\times B_{I}}\right)$$

C_I_: the concentration of metal I in the soil sample,

B_I_: the corresponding background value of metal i.

K was used to correct the regional differences of the soil background value (generally a constant of 1.5). The contamination degree of heavy metals was graded into seven levels (Table 1).

### 2.4. Health Risk Assessment

The HMs of surface soil could enter the human body through dermal absorption, ingestion, and inhalation. Therefore, the average daily doses (ADDs) for ingestion (ADD_ing_), inhalation (ADD_inh_), and dermal (ADD_derm_) exposure pathways (mg/kg·day) were estimated based on the USEPA method as follows (aselineHumanHealt, 2001):

$${\mathrm{ADD}}_{\mathrm{ing}}=\frac{C\times IngR\times EF\times ED\times 10^{-6}}{BW\times AT}$$

$${\mathrm{ADD}}_{inh}=\frac{C\times InhR\times EF\times ED}{PEF\times BW\times AT}$$

$${\mathrm{ADD}}_{\mathrm{derm}}=\frac{C\times SA\times SL\times ABF\times EF\times ED\times 10^{-6}}{BW\times AT}$$

The parameters used in these equations are defined in Table 2.

The non-carcinogenic risk was estimated using a hazard quotient (HQ) for each element and a hazard index (HI) for the sum of HQ through different exposure pathways. HI and HQ were calculated as follows:

HQ = ADD/RfD

HI = ΣHQ

ADD: Daily intake via a certain route, mg/(kg·d);

RfD: Non-carcinogenic reference dose produced via a certain exposure route, mg/(kg·d);

HQ: Hazard quotient;

HI: Hazard index caused by different intake routes.

RfD is the reference dose (mg/kg·day), and the RfD values for each element, and different exposure pathways were obtained from the USEPA Integrated Risk Information System (IRIS).

The risk of cancer due to exposure to HMs was estimated based on Excess Lifetime Cancer Risk (CR). The CR for different exposure pathways was calculated using the following formulas:

CR = ADD × SF

TCR = ΣCR

ADD: Daily intake via a certain route, mg/(kg·d);

SF: Carcinogenic intensity coefficient, (kg·d)/mg;

CR: Carcinogenic risk via a certain exposure route;

TCR: Total carcinogenic risk via different intake routes.

According to the EPA, the reference dose (RfD), skin permeability constant (PC), and carcinogenic intensity factor (SF) of the eight heavy metals in groundwater under the two exposure routes of drinking and skin contact are shown in Table 3.

The evaluation criteria of non-carcinogenic risk and carcinogenic risk are shown in Table 4.

### 2.5. Data Analysis and Statistics

All statistical analyses were performed using Excel 2016 (Microsoft, Redmond, WA, USA). Correlation analysis and principal component analysis were performed to show the relationships between heavy metal contents and soil properties by SPSS 24.0 (IBM, Armonk, NY, USA). Kriging interpolation was applied to determine the spatial distribution of heavy metal content by ArcGIS 10.7 (ESRI, Redlands, CA, USA). In addition, we simulated 10,000 times with a Monte Carlo simulation to identify the risk probability using Crystal Ball v11.0 (Oracle, Austin, TX, USA).

## 3. Results and Discussion

### 3.1. Concentration Characteristics of HMs in Soil under Two Land-Use Types

Table 5 showed the statistical results of eight HMs in soil under two land-use types. The average concentrations of As, no matter which type of land use, lowered the corresponding background values. Cr and Ni slightly exceeded their background value under the two land-use types. The average values of Cd, Hg, and Pb were 9.3, 2.9, and 2.1 times their corresponding background value under irrigated cropland, respectively. In addition, their average values were 20.0, 4.3, and 2.5 times their corresponding background values under industrial land. These results showed that Cd, Hg, and Pb were polluted more seriously in industrial land than in irrigated cropland. Compared with the background values, Cu and Zn were 1.4 and 1.8 times under industrial land, whereas they were 2.5 and 1.6 times under irrigated cropland. This showed that Cu and Zn pollution in irrigated cropland was more serious than in industrial land. This may be caused by the excessive use of pesticides and fertilizers [23]. The coefficients of variation (C.V) of eight heavy metals varied from 33.3% for Ni to 285.7% for Cd under industrial land and decreased in the following order: Cd > Hg > Pb > Cu > Zn > Cr > As > Ni. Additionally, their C.V varied from 37.3% for As to 182.4% for Cu under irrigated cropland, and decreased in the following order: Cu > Cd > Hg > Pb > Cr > Zn > Ni > As. High variability (>0.5) indicated that external factors had a significant effect on the concentrations of HZs in soil. The above results suggest that soils in the study area have been contaminated by HMs to a different degree under different land uses. The results in our study were several times higher than in previous studies, which could be due to the higher levels of HMs in our samples [24,25].

### 3.2. Pollution Evaluation

#### 3.2.1. Index of Geo-Accumulation

**Figure 2:**
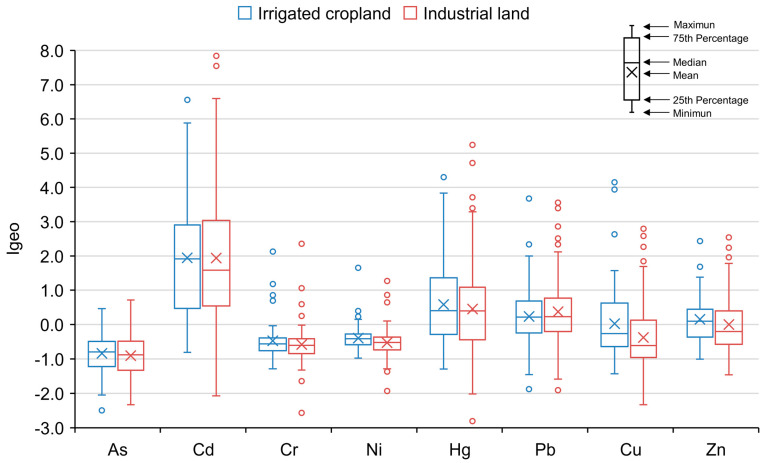
Heavy element boxplots of the index of geo-accumulation under two land covers (irrigated cropland and industrial land).

The Igeo of eight soil heavy metals (As, Cd, Cr, Ni, Hg, Pb, Cu, Zn) in two land uses is presented in Figure 2. The average of Igeo under two land uses decreased in the following order: Cd >Hg > Pb > Zn > Cu > Ni > Cr >As. Compared to the evaluation standard (Table 1), the average of Cu, Ni, Cr, and As was less than zero, which belonged to the no pollution level. The results showed that these four elements were less disturbed by human beings. The average Hg, Pb, and Zn were between 0 and 1, which belonged to the light pollution level. However, the large variation range showed that these three elements were slightly disturbed in some areas. Cd pollution was the most serious and belonged to a slightly moderate pollution level. By comparing land-use types, we found that the pollution of Cd, Hg, and Zn in irrigated cropland was higher than in industrial land, while Pb pollution was higher in industrial land than in irrigated cropland. In summary, Cd is the most serious pollution of the two land-use types.

In irrigated land, Zn pollution might come from the use of chemical fertilizers and the discharge of agricultural wastewater. Although Hg pollution was more serious in irrigated land, the difference was small, which might have been caused by traffic discharge. This finding is consistent with that of Liu, who found that Hg mainly comes from traffic emissions [26]. Both industrial land and irrigation cropland had a similar degree of Cd pollution. A possible explanation for this might be that both industrial and agricultural emissions produce Cd pollution. Our data are consistent with Huang, who reported that Cd pollution was serious in both industrial and agricultural land in China [27]. As Cd is a carcinogenic element, we should focus on its impact on human health. Some reviews have also reported that heavy metal soil contamination in industrial areas was more serious than contamination in agricultural areas. Wang found that the sources of Cd pollution in China were smelting, mining, waste disposal, fertilizer, and pesticide applications, and vehicle exhaust from large to small [28]. These findings suggest that the industrial production process can lead to toxic heavy metal emissions that threaten soil safety. In general, the areas around the industrial facility are being polluted considerably. Therefore, before industrial land was converted into residential land or parkland, a systematic health risk assessment was necessary.

#### 3.2.2. Pollution Distribution

**Figure 3:**
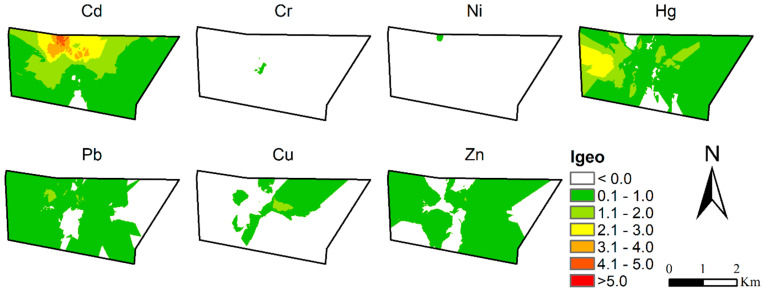
Classification map of heavy metal pollution using the index of geo-accumulation. (Elements without contamination were not displayed).

Figure 3 showed the spatial distribution of the eight heavy metal Igeo in the soil. We found that the pollution areas of Cr and Ni were smaller, and the degree was also lighter. The pollution areas of Pb, Cu, and Zn were relatively large, but they were mainly light pollution degree (0 < Igeo < 1). Hg pollution was mainly light and moderate, and moderate pollution was mainly located on the west side. Cd pollution was the most serious. Highly polluted areas were mainly located on the north side, and some areas have reached a serious level and should be paid enough attention to. In summary, Cd and Hg had the most serious pollution, with high pollution areas located on the north and west sides, respectively.

The spatial distribution of heavy metals indicates the dominant role of industrial activities as major pollution sources. The industries near the Cd pollution site mainly included a sludge landfill, thermal power plant, and spice plant, and the Hg pollution site included a sludge landfill, wastewater treatment, and an ink plant. The industrial production process can lead to extensive emissions of dust and solid wastes containing toxic heavy metals, which are significant sources of soil contamination. High concentrations of heavy metals, such as Cd, Cr, Cu, Pb, and Zn, have been found in soils around the old industrial city in different regions [29]. Therefore, the spatial distribution of heavy metals also reflected the extent and degree of contamination that originated directly from industrial activities in Putuo.

### 3.3. Source Analysis

#### 3.3.1. Correlation Analysis

**Figure 4:**
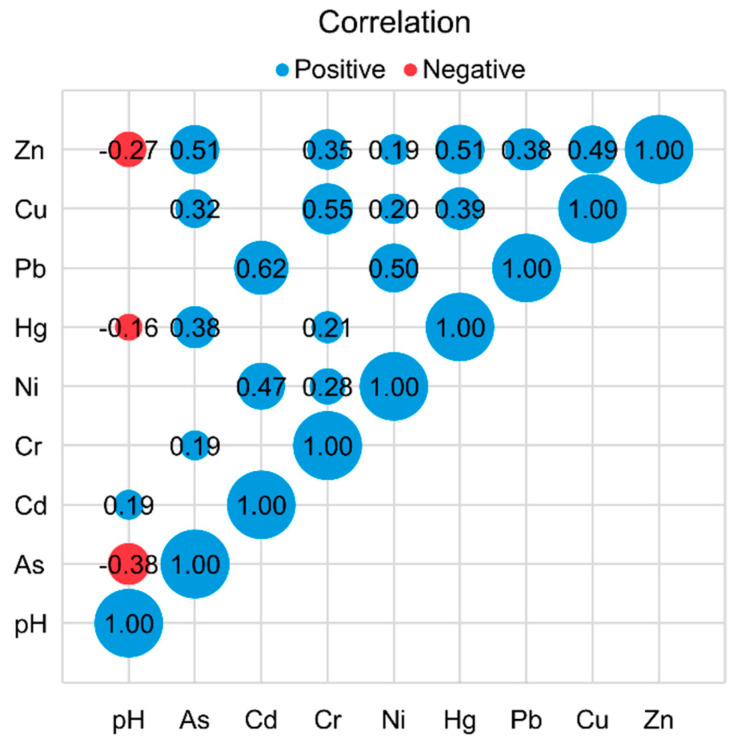
The correlations between heavy metals by combining Pearson correlation analysis (no significance had not been shown).

The Pearson correlation coefficients between heavy metals in soil are presented in Figure 4. A positive correlation between heavy metals in soil suggests that they probably have the same pollution source. A significant correlation was found between As–Zn, Cd–Pb, Cr–Cu, Ni–Pb, Hg–Zn, and Cu–Zn (*p* < 0.01). Meanwhile, pH presented a significant negative correlation with As, Hg, and Zn (*p* < 0.01). This might be due to the reason that these three elements are more easily dissolved at higher pH and flow away with rainfall, resulting in lower concentrations in the soil. As, Pb, Cd, and Zn may come from human influence, such as traffic emissions and industrial activities [30]. Many other studies have also reported that natural sources (such as base metal and diagenetic sources) are the main causes of Cr and Ni [31]. Previous studies believe that the enrichment of these elements mainly comes from human activities [32]. To better reveal the correlation among the soil heavy metals and quantify their sources, PCA was applied in this study.

#### 3.3.2. Principal Component Analysis Based on Land-Use Covers

To explore the sources of eight heavy metals in the soil of the study area, the source analysis was carried out by using principal component analysis, and the results are shown in Table 6 and Table 7 The value of KMO (Kaiser-Meyer-Olkin Measure of Sampling Adequacy) in industrial land and irrigation cropland was 0.551 and 0.739, respectively, both higher than 0.5, which met the premise of the PCA application. In industrial land soil, we found that the first principal component (PC1) was closely related to As, Hg, Cu, and Zn and explained 28.468% of the total variation. The second principal component (PC2) included Cd, Ni, and Pb, whereas Cr was classified as the third principal component (PC3). PC2 and PC3 contributed 25.719% and 13.073% of the total variation, respectively. Similarly, in irrigation cropland soil, PC1 included As, Cr, Hg, Cu, and Zn, whereas Cd, Ni, and Pb were classified as PC2. PC1 and PC2 contributed 44.081% and 30.978% of the total variation, respectively. In summary, heavy metals in soil can be classified into three components in industrial land, and two components in irrigation cropland.

Eight heavy metals in industrial land soil were divided into three categories. PC1 included As, Hg, Cu, and Zn, which could be defined as industrial sources. Anthropogenic industrial activities have produced large quantities of wastewater and gas that contain many heavy metals. These pollutants enter the surrounding soils with surface runoff, rainwater leaching, and atmospheric deposition [33]. PC2, including Cd, Ni, and Pb, could be considered an anthropogenic component correlated with traffic activities. Pb pollution in soil is highly correlated with traffic emissions and can be regarded as a symbolic element polluted by motor vehicles. This conclusion has been confirmed by many relevant studies over the past 50 years [34,35]. PC3 could be classified as a natural source because the average values of Cr were lower than the background concentrations. Moreover, as shown in Figure 2, the concentration distribution of Cr was concentrated. This result showed that Cr was less disturbed by humans. This result is consistent with that of Li, who found that the contents and spatial distribution of Cr are mainly controlled by natural factors [36].

**Figure 5:**
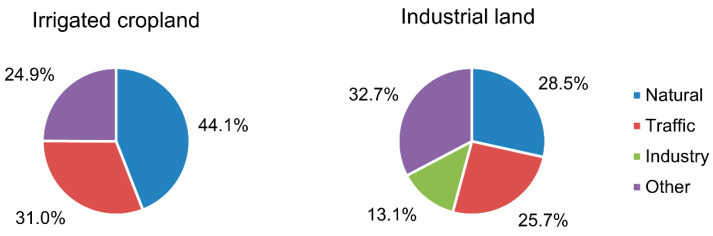
Pollution sources of heavy metals in soil using the PCA model.

Eight heavy metals in irrigation cropland soil were divided into two categories. PC1 included As, Cr, Hg, Cu, and Zn, and could be defined as agricultural sources. Cu mainly comes from the application of pesticides and chemical fertilizers [37]. PC2 included Cd, Ni, and Pb, and could be defined as the traffic source. Pb is a characteristic pollutant in traffic activities [38]. For the cumulative contribution under the two land types, we found that the two principal components under irrigation cropland accounted for 75.059% of the total variation, while the three principal components under industrial land explained only 67.26% of the total variation. This result showed that the sources of heavy metals in industrial land were more complex. Figure 5 showed the sources of eight heavy metals under two land-use types. Under industrial land, other sources accounted for 32.7%. This showed that the pollution sources under industrial land were more complex than under irrigated cropland.

### 3.4. Health Risk Assessment Based on Land-Use Covers

#### 3.4.1. Identify the Main Pollution Routes

**Figure 6:**
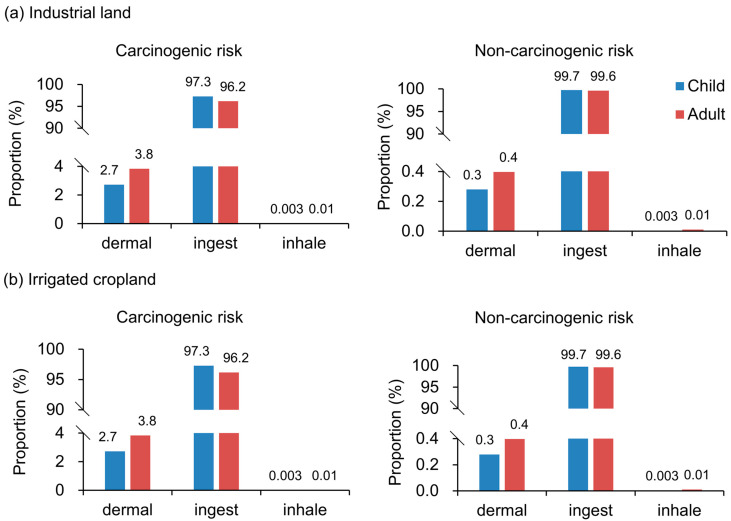
Risk differences of three exposure routes (ingest, dermal, and inhale) in adults and children. (**a**) Industrial land, (**b**) Irrigated cropland.

Figure 6 showed the risk proportion of three pollution routes under two land-use covers. We found that ingestion risks were dramatically higher than dermal exposure and inhale exposure. Under industrial land, the proportion of ingest in carcinogenic risk for children and adults was 97.3% and 96.2%, respectively. Under industrial land, the proportion of ingest in non-carcinogenic risk for children and adults was 99.7% and 99.6%, respectively. In irrigated croplands, the risk proportion of intake was far greater than that of the other two pollution routes. Notably, children had a higher risk proportion of ingesting than adults. Consequently, the results showed that ingest was the major risk-exposure method.

This result is consistent with that of Varol et al., who also found that ingest was the riskiest route [39]. Due to their physiological characteristics, such as hand-finger sucking, which has been generally regarded as one of the key exposure pathways of soil metals for children [40], children are more susceptible to a given dose of toxin and are likely to inadvertently ingest significant quantities of soil.

#### 3.4.2. Identify Priority Control Pollutants

**Figure 7:**
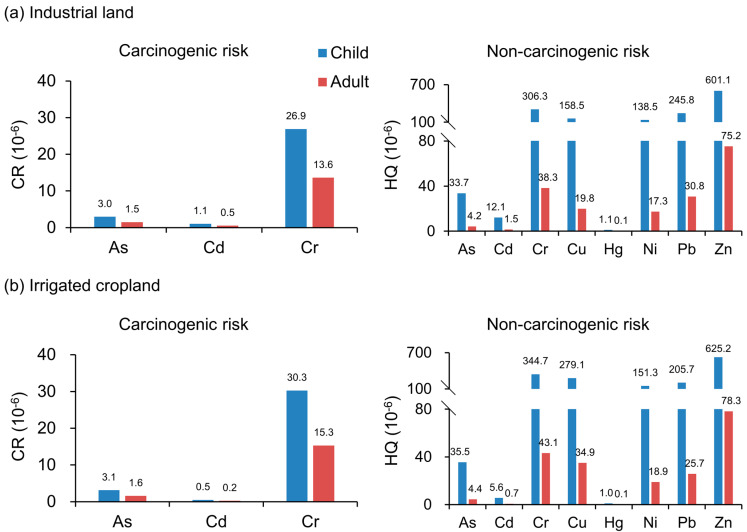
Health risk of different elements risk in children and adults under two land-use types. (**a**) Industrial land, (**b**) Irrigated cropland.

Figure 7 shows the carcinogenic and non-carcinogenic risks of adults and children under two land-use types. We found that Cr had the highest carcinogenic risk under the two groups of land-use types, and children had a higher risk than adults. Compared with Table 4, we found that no elements had a significant carcinogenic risk (>10^−4^). As and Cr had an acceptable carcinogenic risk (10^−6^–10^−4^). Cd was safe under irrigated cropland, but children had an acceptable carcinogenic risk under industrial land. In addition, Cr, Cu, Ni, Pb, and Zn had a higher non-carcinogenic risk and a far greater impact on children than adults, especially Zn. However, compared with Table 4, we found that the non-carcinogenic risks of the eight elements were safe. In summary, non-carcinogenic risks should focus on Zn, and carcinogenic risks should focus on Cr.

These results are in line with the findings of a study on Lake Urmia in Iran. Multiple HM exposures could lead to additive, synergistic, or antagonistic health effects in the human body [41]. These results are also consistent with the findings of Zhang et al. [42], who reported that, as a result of intensifying economic activities, environmental pollution and related health effects are often concentrated in areas with rich resources of Cr. By comparing the non-carcinogenic and carcinogenic risk values for children and adults, it can be concluded that children are at a higher risk of suffering adverse health effects from metal exposures than adults. Additional ingestion pathways, such as pica behavior and hand or finger sucking, are known to contribute to heightened HI and TCR risks in children [43].

#### 3.4.3. Identify Priority Control Areas

(1) Non-carcinogenic risk

**Figure 8:**
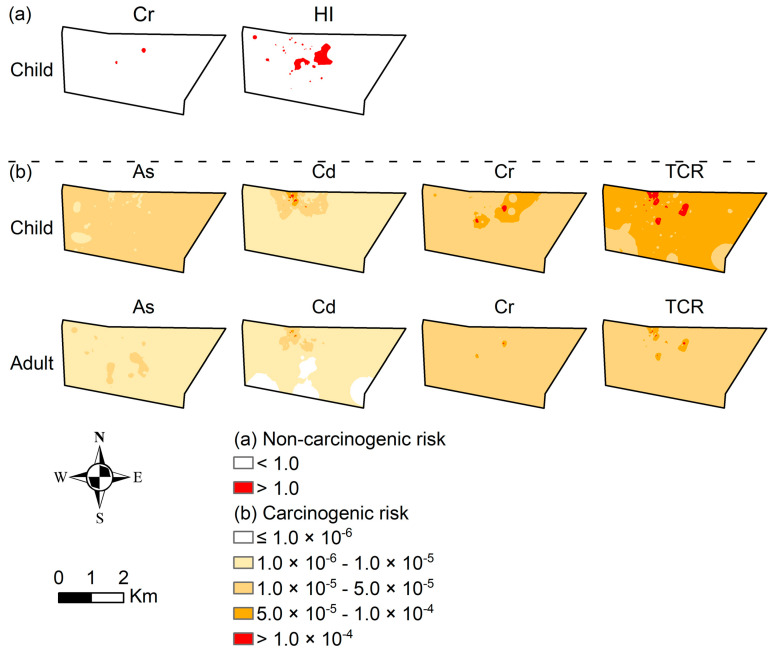
Health risks of heavy metal in soil for children and adults. (**a**) Non-carcinogenic risk; (**b**) Carcinogenic risk. (Elements without risk were not displayed).

Figure 8 showed the spatial characteristics of health risks using Kriging interpolation. We found that Cr had a significant non-carcinogenic risk for children in two central regions. Overall, the non-carcinogenic risk to children was mainly distributed in the middle. However, we did not find that adults suffered a non-carcinogenic risk, indicating that children were more sensitive than adults. Table 8 showed the area proportion of eight elements of non-carcinogenic risk. We found that the non-carcinogenic hazard was mainly the effect of Cr on children, and the area of significant non-carcinogenic risk (>1) accounted for 0.2%. The significant non-carcinogenic risk area of HI was 4.1%. In summary, the non-carcinogenic risk was mainly manifested by the impact of Cr on children, and the high-risk areas were located in the central part of the study area. Therefore, the government should pay more attention to these highly polluted areas and adopt soil remediation technologies to reduce environmental hazards.

(2) Carcinogenic risk

The spatial distribution of the risk levels of the three carcinogenic elements is shown in Figure 8. We found that the significant carcinogenic risk (>10^−4^) of Cd was mainly located in the north of the study area. Cr has two major carcinogenic risk areas located in the central and northern regions. The spatial distribution of the TCR showed that the high-risk areas showed a punctate distribution, mainly located on the north side, and that the risk of children was higher than adults. Table 9 showed the area proportion of different risk levels. The significant carcinogenic risk area to children caused by the three carcinogenic elements reduced in the following order: Cr (0.3%) > Cd (0.1%) > As (0.0%). In summary, the high-risk area was mainly located in the north of the study area.

#### 3.4.4. Cumulative Risks

**Figure 9:**
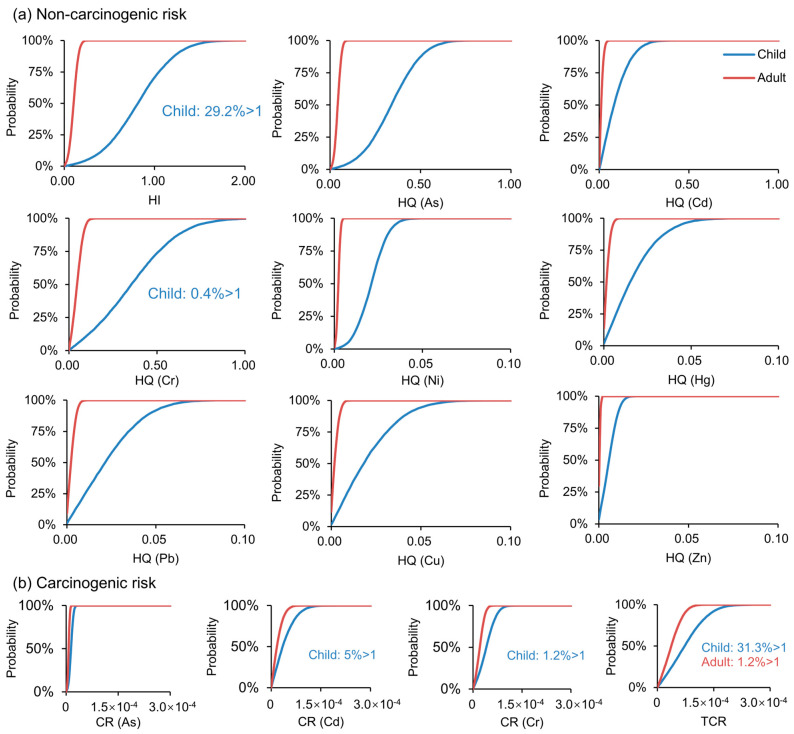
Cumulative probability of non-carcinogenic and carcinogenic risks. (**a**) Non-carcinogenic risk, (**b**) Carcinogenic risk.

The health risk results of soil exposure to eight heavy metals based on Monte Carlo simulation are shown in Figure 9. We found that the probability of Cr exceeding the non-carcinogenic risk threshold (>1) was 0.4% for the child. The risk probability of the hazard index for children was 29.2%. However, the eight heavy metals had no possibility of non-carcinogenic risks to adults. The probability of Cd and Cr exceeding the carcinogenic risk threshold (>10^−4^) was 5% and 1.2% for the child, respectively. Meanwhile, the three carcinogenic elements had no possibility of significant carcinogenic risk (>10^−4^) to adults. In summary, the probability that soil exposure produced health risks for children was much higher than for adults. In addition, we should focus on the carcinogenic risk of Cd to children.

Therefore, we suggest that the government should pay more attention to children’s health risks and take measures to reduce children’s exposure time.

The major strength of this study was its determination of the sources and distribution of pollution. However, our study has two limitations. In this study, only the total concentration of each element was measured. However, the toxicity of the metal was related to its form and valence state. For example, inorganic As is the most toxic form of As, and Cr (VI) is a toxic form of this metal. However, our study did not consider the valence state of heavy metals, which may have influenced the risk results. Although our research content was limited to specific cases, the results suggest that industrial land has higher health hazards to soil than irrigation land. This should arouse sufficient attention to industrial pollution. Additional studies are needed to determine the relationship between land-use types and human health to safely plan urban development.

## 4. Conclusions

This study assessed the contamination levels, sources, pollution distribution, and health risks of eight typical heavy metals in the industrial area soils of east China. Igeo showed that Cd, Hg, and Pb had the most serious pollution under the two land-use types. PCA identified the pollution sources under two land-use types: under irrigated cropland, the main sources were natural sources and traffic sources; under industrial land, the main sources were natural sources, industrial sources, and traffic sources. The health risk assessment model showed that ingest was the main pollution route, Cr and Zn were high pollution factors of carcinogenic risk and non-carcinogenic risk, respectively, and the high-risk area was mainly located north of the study area. Therefore, it is necessary to take effective measures in future industrial and agricultural production activities to ensure sustainable economic development. Specific measures or policies are as follows: (1) reduce the input of chemical fertilizers and pesticides; (2) strictly limit the discharge of industrial waste; (3) improve the emission standard of automobile exhaust; (4) prohibit the conversion of land in high-risk areas into residential land; (5) maintain a certain safe distance between high-risk areas and crowd. This study suggests that the government should pay more attention to the health risks of heavy metals in the soil of industrial areas.

## Figures and Tables

**Table 1:** Criteria for geo-accumulation index.

Igeo	Grades	Pollution Level
Igeo ≤ 0	0	No
0 < Igeo ≤ 1	1	Light
1 < Igeo ≤ 2	2	Little moderate
2 < Igeo ≤ 3	3	Moderate
3 < Igeo ≤ 4	4	Little heavy
4 < Igeo ≤ 5	5	Heavy
Igeo ≥ 5	6	Severe

**Table 2:** Parameter model of soil health risk assessment.

Parameters	Description	Units	Adult	Child
ADD_ing_	daily intake via ingest	mg/(kg·d)	-	-
ADD_inh_	daily intake via inhale	mg/(kg·d)	-	-
ADD_derm_	daily intake via dermal	mg/(kg·d)	-	-
C	Metal concentration	mg/kg	-	-
IngR	Ingestion rate of soil	mg/d	100	200
InhR	Inhalation rate of soil	m^3^/d	15	7.5
EF	Exposure frequency	d/a	350	350
ED	Exposure duration	A	24	6
BW	Body weight	kg	60	15
AT (Non-carcinogens)	Average time	d	24 × 365	6 × 365
AT (Carcinogens)	Average time	d	70 × 365	70 × 365
PEF	Particle emission factor	m^3^/kg	1.36 × 10^9^	1.36 × 10^9^
SA	Surface area	cm^2^/d	5700	2800
SL	Skin adherence factor	mg/cm^2^	0.07	0.2
ABF (Non-carcinogens)	dermal adsorption factor	-	0.001	0.001
ABF (Carcinogens)	dermal adsorption factor	-	0.01	0.01

**Table 3:** Soil parameter values of SF and RFD.

		As	Cd	Cr	Ni	Hg	Pb	Cu	Zn
RfD	ingest	3 × 10^−4^	1 × 10^−3^	3 × 10^−3^	0.02	3 × 10^−4^	3.5 × 10^−2^	0.04	0.3
	dermal	3 × 10^−4^	1 × 10^−5^	6 × 10^−5^	5.4 × 10^−3^	2.4 × 10^−5^	3.5 × 10^−2^	0.04	0.3
	inhale	1.23 × 10^−4^	1 × 10^−3^	2.86 × 10^−5^	2.06 × 10^−2^	3 × 10^−4^	5.25 × 10^−4^	0.012	0.06
SF	ingest	1.5	6.1	0.5					
	dermal	3.66	6.1	-					
	inhale	15.1	1.8 × 10^−3^	42					

**Table 4:** Health risk assessment criteria.

Extent of Risk	HQ or HI	CR or TCR
safe	≤1	≤1 × 10^−6^
acceptable risk		1 × 10^−6^–1 × 10^−4^
significant risk	>1	>1 × 10^−4^

**Table 5:** Statistical results of heavy metal concentrations in soil (mg/kg).

Element	Industrial Land (*n* = 140)					Irrigated Cropland (*n* = 53)					Background ^a^
	Min	Max	Mean	SD	C.V%	Min	Max	Mean	SD	C.V%	
As	2.7	22.3	7.9	3.3	41.8	2.4	18.7	8.3	3.1	37.3	9.05
Cd	0.1	50.1	2.8	8.0	285.7	0.1	12.4	1.3	2.2	169.2	0.14
Cr	16.9	512.0	71.7	42.9	59.8	41.1	437.0	80.7	58.6	72.6	66.8
Ni	11.8	108.0	32.4	10.8	33.3	22.8	141.0	35.4	16.1	45.5	29.9
Hg	0.0	4.0	0.3	0.5	166.7	0.0	2.1	0.2	0.3	150.0	0.07
Pb	9.2	406.0	57.5	57.5	100.0	9.4	441.0	48.1	57.7	120.0	23.0
Cu	7.7	268.0	37.1	35.2	94.9	14.4	686.0	65.3	119.1	182.4	25.8
Zn	42.5	681.0	140.6	109.9	78.2	58.2	631.0	146.3	97.5	66.6	78.0

^a^ Background value of soil in Shanghai (1990).

**Table 6:** Principle component analysis of heavy metals in industrial land soil.

Element	Component		
	PC 1	PC 2	PC 3
As	**0.724**	−0.088	0.001
Cd	−0.104	**0.922**	−0.062
Cr	0.089	0.053	**0.965**
Ni	0.029	0.701	0.299
Hg	**0.746**	0.001	−0.012
Pb	0.301	**0.806**	−0.072
Cu	**0.663**	0.119	0.092
Zn	**0.804**	0.206	0.089
Eigenvalues	2.277	2.058	1.046
% of variance	28.468	25.719	13.073
% of Cumulative	28.468	54.187	67.26

Significant loading (>0.6) is in bold.

**Table 7:** Principle component analysis of heavy metals in irrigation cropland soil.

Element	Component	
	PC 1	PC 2
As	**0.687**	−0.09
Cd	0.033	**0.81**
Cr	**0.873**	0.27
Ni	0.157	**0.905**
Hg	**0.826**	0.021
Pb	0.044	**0.941**
Cu	**0.905**	0.091
Zn	**0.874**	0.168
Eigenvalues	3.526	2.478
% of variance	44.081	30.978
% of Cumulative	44.081	75.059

Significant loading (>0.6) is in bold.

**Table 8:** Area proportion of non-carcinogenic risk.

Groups	Range	Area Proportion		
		HQ (Seven Other Elements)	HQ (Cr)	HI
Child	<1	100.0%	99.8%	95.9%
	>1	0.0%	0.2%	4.1%
Adult	<1	100.0%	100.0%	100.0%
	>1	0.0%	0.0%	100.0%

**Table 9:** Area proportion of carcinogenic risk.

Groups	Range	Area Proportion			
		CR (As)	CR (Cd)	CR (Cr)	TCR
Child	<10^−6^	0.0%	0.0%	0.0%	0.0%
	10^−6^–10^−5^	4.2%	89.5%	0.0%	0.0%
	10^−5^–5 × 10^−5^	95.8%	9.4%	90.4%	13.3%
	5 × 10^−5^–10^−4^	0.0%	1.0%	9.3%	84.4%
	>10^−4^	0.0%	0.1%	0.3%	2.2%
Adult	<10^−6^	0.0%	13.7%	0.0%	0.0%
	10^−6^–10^−5^	95.5%	81.8%	0.0%	0.0%
	10^−5^–5 × 10^−5^	4.5%	4.3%	99.6%	97.6%
	5 × 10^−5^–10^−4^	0.0%	0.1%	0.3%	2.3%
	>10^−4^	0.0%	0.0%	0.0%	0.1%

## Data Availability

The data presented in this study are available on request from the corresponding author. The data are not publicly available due to privacy.
